# Morphological and Molecular Phylogeny of Two New Ciliate Species, *Colpoda heilongjiangensis* n. sp. and *Bryometopus shii* n. sp. (Protozoa, Ciliophora, Colpodea), from Northeastern China

**DOI:** 10.3390/microorganisms14051034

**Published:** 2026-05-01

**Authors:** Qiyue Zhao, Jiatong Guo, Menghan Liu, Bojie Yin, Bailin Li, Yumeng Song, Xuming Pan

**Affiliations:** 1 Laboratory of Protozoology, Harbin Normal University, Harbin 150025, China; 2 Key Laboratory of Biodiversity of Aquatic Organisms, College of Life Science and Technology, Harbin Normal University, Harbin 150025, China

**Keywords:** taxonomy, new species, SSU-rRNA genes, phylogeny, ciliates

## Abstract

This study describes two new species of ciliates, *Colpoda heilongjiangensis* n. sp. and *Bryometopus shii* n. sp., discovered in northeastern China. *Colpoda heilongjiangensis* n. sp. was collected from a sewage outlet in Harbin, characterized by its large size, broadly to slenderly reniform body shape, and unique ciliary patterns. *Bryometopus shii* n. sp. was found in a puddle near a waste disposal station, featuring a droplet-shaped or oval and distinct oral apparatus. The morphology of both species was investigated through live observation, protargol staining, and silver carbonate and dry silver nitrate impregnation. DNA extraction and SSU-rRNA gene sequencing were performed to determine their evolutionary relationships. Phylogenetic analyses based on SSU-rRNA gene data revealed that *Colpoda heilongjiangensis* n. sp. clusters with *C. minima*, while *Bryometopus shii* n. sp. has a close relationship with *B. atypicus*. This study also discussed the non-monophyletic status of the genus *Bryometopus* and proposed a revision to the family Tillinidae based on morphological and molecular evidence. These findings highlight the remarkable diversity of Colpodea and contribute to the understanding of their evolutionary relationships.

## 1. Introduction

Ciliates in the class Colpodea Small & Lynn, 1981, commonly found in ecosystems worldwide, exhibit a great biological diversity and play important roles in terrestrial and water environments [1,2,3,4,5,6,7,8,9,10,11,12,13,14,15]. However, due to their small body size and a high degree of similarity in ciliature, the taxonomy of this group of organisms remains difficult and confusing [16,17,18,19]. Recent studies across China have revealed considerable species diversity within the class Colpodea ciliates, and the continuous discovery of new taxa underscores an urgent need for further systematic research on this protozoan group [20,21]. The class Colpodea is made up of typical soil-dwelling ciliates, comprising about 60 genera and more than 200 species [22,23]. Foissner divided the class Colpodeas into two subclasses, Colpodia Foissner, 1985 and Bryometopia Foissner, 1985, based on the characteristics of the reticulate silverline system [24,25,26,27,28,29].

As part of an ongoing faunistic study of freshwater ciliates in northeastern China, the morphology and phylogeny of two species in the class Colpodea, *Colpoda heilongjiangensis* n. sp. and *Bryometopus shii* n. sp., are investigated based on living observation and protargol, silver carbonate and silver nitrate staining. Both species were sampled within the framework of this long-term systematic investigation. Based on their unique infraciliature, especially with respect to the oral apparatus, these two forms are regarded as new taxa at the species level. Molecular phylogenies of two new species based on SSU-rRNA sequence data were carried out to determine their evolutionary relationships.

## 2. Material and Methods

### 2.1. Sample Collection and Identification

*Colpoda heilongjiangensis* sp. nov. was collected on 14 May 2024 from a sewage outlet near a gas station in Hulan District, Harbin, Heilongjiang Province, Northeast China (45°57′10″ N, 126°35′12″ E). At the sampling site, the water temperature was 12 °C and the pH value was 7.2. *Bryometopus shii* sp. nov. was collected on 14 May 2024 from a puddle adjacent to the waste disposal station in Xizha Village, Harbin, Heilongjiang Province, Northeast China (45°40′27″ N, 126°43′23″ E). The water temperature at the sampling site was 13 °C, with a pH of 7.0.

Approximately 0.5 L of water was gathered from depths ranging between 0.1 and 0.5 m beneath the sampling site using a sterile collection bottle. After the samples were returned to the laboratory, the ciliates were isolated from the collected samples. The raw environmental samples were additionally screened, and only several euplotid ciliates were detected. At room temperature (about 24 °C), raw cultures were established in Petri dishes containing filtered habitat water. Sterilized rice grains were added to nourish bacterial growth, serving as a food source for the ciliates.

Living specimens were observed and photographed in vivo using bright field and differential interference contrast microscopy (Zeiss Axio Imager A2, Zeiss, Oberkochen, Germany). The protargol staining and silver carbonate staining techniques were used to reveal the ciliary pattern [30]. The dry silver nitrate impregnation method was employed to reveal its silverline system [31,32]. Measurements were taken at magnifications of ×100–1000, and drawings of stained specimens were created utilizing a drawing apparatus. The final plates were prepared using Adobe Photoshop 3. Classification and terminology are mainly based on Foissner [16] and Lynn [33].

### 2.2. DNA Extraction, PCR Amplification and Sequencing

Three to five cells of *Colpoda heilongjiangensis* n. sp. and *Bryometopus shii* n. sp. were isolated from the clonal cultures, respectively, and washed with distilled water (at least three times) to eliminate residual contaminants. After 6 h maintenance in non-nutrient distilled water, the cells were transferred to a 1.5 mL microcentrifuge tube (Eppendorf AG, Hamburg, Germany) containing up to 5 μL of distilled water. Total genomic DNA was extracted using the DNeasy Blood & Tissue Kit (Qiagen, Hilden, Germany) according to the manufacturer’s protocol. DNA concentration was quantified via NanoDrop spectrophotometry. The SSU-rRNA gene was amplified with universal eukaryotic primers EukA (5′-AAC CTG GTT GAT CCT GCC AGT-3′) and EukB (5′-TGA TCC TTC GTC AGG TTC ACC TAC-3′) [34]. The following PCR conditions were as follows: initial denaturation at 94 °C for 5 min; 5 cycles at 94 °C for 30 s, 56 °C for 105 s, and 72 °C for 120 s; 25 cycles at 94 °C for 45 s, 60 °C for 105 s, and 72 °C for 120 s; and a final extension at 72 °C for 8 min [35]. For each gene, three positive clones were selected and sent to Sangon Biotech (Shanghai, China) for bidirectional sequencing. Only one sequence was retained for subsequent data analysis. A total of two new SSU-rRNA gene sequences of Colpodea were determined.

### 2.3. Phylogenetic Analyses

In addition to the two newly sequenced species, 73 SSU-rRNA gene sequences downloaded from the GenBank database for phylogenetic analyses, included three species of tetrahymenid taxa (*Tetrahymena pyriformis*, *T*. *tropicalis* and *T*. *americanis*) as the outgroup. The SSU-rRNA gene sequences were aligned using the ClustalW 1.8 algorithm implemented in BioEdit 7.0.1 [36], followed by manual curation of ambiguously aligned regions within the software environment, resulting in a final alignment of 1770 positions. Phylogenetic trees were reconstructed using maximum likelihood (ML) implemented in RAxML-HPC2 v8.2.12 [37] and Bayesian inference (BI) performed with MrBayes v3.2.7a [38], both executed through the CIPRES Science Gateway platform [39]. The optimal substitution model for SSU-rRNA gene phylogenetics was determined as GTR + I + G using Modeltest v3.4. The MrBayes settings were as follows: nst = 6 (for GTR) and rates = invgamma [40]. The MCMC analyses used four chains (one cold, three heated; temp = 0.2) and ran for 10 million generations, with sampling every 1000 generations. The first 25% of samples were discarded as burn-in. Convergence was confirmed when the average standard deviation of split frequencies < 0.01 and ESS > 200 for all parameters in Tracer. A support value < 70%/0.70 (ML/BI) was considered as low, 70%/0.70 (ML/BI)–94%/0.94 (ML/BI) as moderate, and >95%/0.95 (ML/BI) as high. Final phylogenetic trees were annotated and visualized in MEGA 7.0 [41].

## 3. Results

**Colpodea Small and Lynn, 1981**

**Colpodida Puytorac et al., 1974**

**Colpodidae Bory De St. Vincent, 1826**

***Colpoda*** **Müller, 1773**

**Figure 1:**
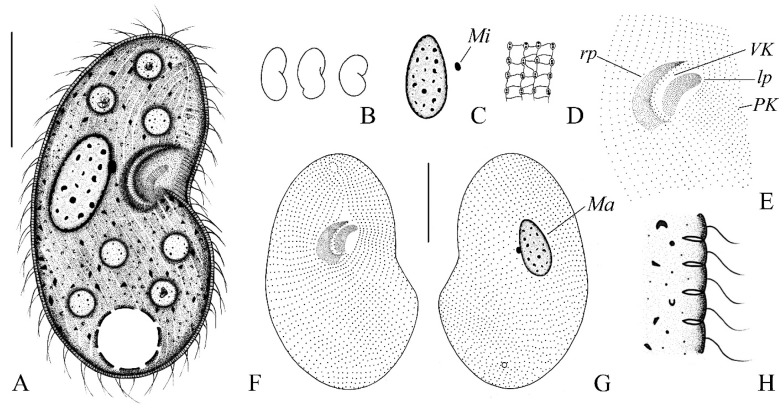
(**A**–**F**) Morphology and ciliary pattern of *Colpoda heilongjiangensis* n. sp. in vivo (**A**,**B**,**H**), after protargol staining (**C**,**E**–**G**) and after silver nitrate impregnation (**D**). (**A**) Ventral view of a representative specimen. (**B**) Different body shapes. (**C**) Macronucleus and micronucleus. (**D**) Silverline system. (**E**) Details of the oral apparatus. (**F**,**G**) Ventral (**F**) and dorsal (**G**) views of the holotype specimen showing the ciliary pattern and the macronucleus. (**H**) Part of pellicle, to show extrusomes. Ma, macronucleus; Mi, micronucleus; VK, vestibular kineties; PK, postoral kineties; rp, right polykinetid; lp, left polykinetid. Bars, 75 μm (**A**,**F**,**G**).

***Colpoda heilongjiangensis* n. sp. (Figure 1A–H, Figure 3A–K and Figure 4A–H; Table 1)**

**Diagnosis.** Body 200–260 × 120–180 μm in vivo; broadly to slenderly reniform in outline; cytoplasm colorless with several minute crystals; cortical granules gray; single macronucleus; 56–67 somatic kineties; 14–17 postoral kineties; 3–5 vestibular kineties; single macronucleus; freshwater habitat.

**Type locality.** A sewage outlet in Harbin Hulan District gas station, Harbin, Heilongjiang, northeastern China (45°57′10″ N; 126°35′12″ E; Figure 1).

**Type material.** The protargol staining holotype specimen (Figure 1F,G and Figure 3A,B) marked with an ink circle has been deposited in the Laboratory of Protozoology, Ocean University of China (slide number: ZQY-2025072701). A paratype slide (registration number: ZQY-2025072701-02) with protargol staining is deposited in the Laboratory of Protozoology, Harbin Normal University.

**Etymology.** The species-group name ‘*heilongjiangensis*’ indicates the area (Heilongjiang) from which the species was collected.

**Figure 2:**
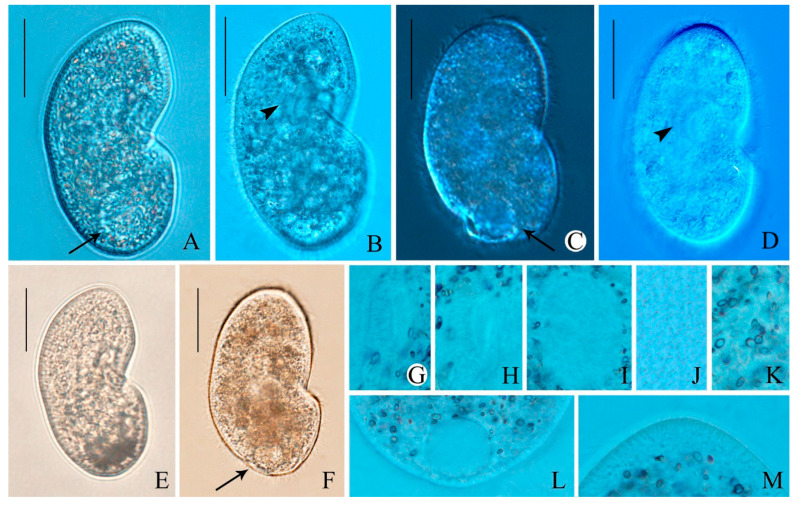
(**A**–**L**) Photomicrographs of *Colpoda heilongjiangensis* n. sp. from life. (**A**–**F**) Right lateral views of different representative cells; arrows in (**A**,**C**,**F**) show contractile vacuole; arrowheads in (**B**,**D**) show oral apparatus. (**G**–**M**) Details of the cell, (**G**,**H**) showing adoral membranelles, (**I**) showing the macronucleus, (**J**) showing refringent granules, (**K**) showing elliptical crystals, (**L**) showing contractile vacuole, and (**M**) showing extrusome. Bars, 75 μm (**A**–**F**).

**Figure 3:**
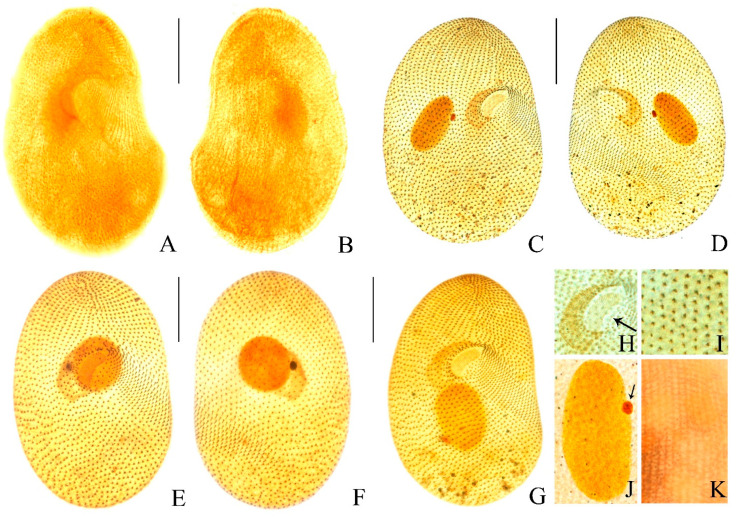
(**A**–**K**) Photomicrographs of *Colpoda heilongjiangensis* n. sp. after protargol staining (**A**,**B**), silver carbonate-staining (**C**–**J**) and dry silver nitrate impregnation (**K**). (**A**,**B**) Ventral and dorsal views of the holotype specimen after protargol staining. (**C**–**G**) Paratype specimens, showing the ciliature on ventral and dorsal views after silver carbonate-staining. (**H**) Oral apparatus, arrow showing left polykinetid. (**I**) Somatic kineties, composed of dikinetids. (**J**) Nuclear apparatus; micronucleus (arrow). (**K**) Silverline system. Bars, 75 μm (**A**–**G**).

**Morphology.** Size in vivo about 200–260 × 120–180 μm, flexible, broadly to slenderly reniform in outline; diagonal groove deep, single postoral sack less noticeable (Figure 1A,B and Figure 2A–F). Cytoplasm colorless with gray cortical granules about 2–3 μm, containing several slightly yellowish minute crystals concentrated throughout the cell. Cells brown-black under low magnification due to numerous anisotropic substances concentrated in the vicinity of the contractile vacuole (Figure 2A,F,K). Opaque cortical layer containing extrusomes about 2 μm thick; extrusomes in vivo inconspicuous, rod-shaped, about 1.5 × 0.3 μm (Figure 1A,H and Figure 3B,E,F,M). Contractile vacuole was slightly ahead of posterior end, approximately 32–35 μm in diameter during diastole, without canals (Figure 1A and Figure 2A,C,F,L). Single excretory pore and cytopyge near center of posterior pole (Figure 1G and Figure 3B,D,F). Macronucleus circular to ellipsoid, about 1/4 of body length, usually located in the mid-body region between the vestibulum and dorsal side, approximately 55 × 35 μm (Figure 1A,C,G, Figure 2I and Figure 3A–G,J). Single micronucleus about 7 × 5 μm, adjacent to macronucleus (Figure 1A,C,G, Figure 2I and Figure 3A–G,J). Silverline system cucullus-type (Figure 1D and Figure 3K). Cilia approximately 9 μm long, closely packed (Figure 1A and Figure 2A–F). Movement occurs through moderate-speed rotational motion around its longitudinal axis, often adhering to debris or remaining stationary at the bottom of the culture dish.

Somatic kineties strongly spirally coursed, ranging in number from 56 to 67 (Figure 1A,F,G, Figure 2B–D,F and Figure 3A–G,I). Dikinetids highly arranged in diagonal groove, but arranged erratically on rear postoral sack. Kineties on the right side extend nearly parallel to the straight preoral suture and longitudinal body axis, respectively (Figure 1G and Figure 3B,D,F). About 14–17 postoral and 3–5 vestibular kineties (Figure 1E,F,G and Figure 3A–G). Macronucleus approximately 50 × 35 μm. Single micronucleus about 7 × 5 μm, adjacent to macronucleus (Figure 1C,G and Figure 3A–G,J). Silverline system cucullus-type (Figure 1D and Figure 3K).

Oral apparatus slightly above the body’s midsection, close to the equatorial region. Two-thirds of oral polykinetids located within horn-shaped vestibulum, parallel to each other and the body’s long axis (Figure 1A and Figure 3A,B,D). Left polykinetid moon-shaped, comprising about 32–36 kineties. Right oral polykinetid proximal portion narrowed and curved hook-like towards the ventral side, basal bodies irregularly arranged (Figure 1E,F, Figure 2B,D,G,H and Figure 3A,C,E,G,H).

**Table 1:** Morphometric characterization of *Colpoda heilongjiangensis* n. sp. based on protargol-stained specimens.

Character	Min	Max	Mean	M	SD	CV	n
Body length, μm	205	259	233.4	239.6	16.1	6.9	25
Body width, μm	130	173	159.8	163.4	11.7	7.3	25
Macronucleus, number	1.0	1.0	1.0	1.0	0.0	0.0	25
Macronucleus length, μm	39	69	54.7	56.3	9.2	16.9	25
Macronucleus width, μm	29	45	36.8	36.4	4.8	13.0	25
Micronucleus, number	1.0	1.0	1.0	1.0	0.0	0.0	25
Micronucleus length, μm	6	9	7.6	7.5	0.9	12.0	25
Micronucleus width, μm	4	5	4.6	4.4	0.5	10.5	25
Distance from anterior end to distal edge of vestibulum, μm	50	75	59.7	56.9	7.5	12.6	25
Distance from anterior end to proximal edge of vestibulum, μm	79	115	95.2	95.5	11.9	12.5	25
Somatic kineties, number	56	67	60.9	60.0	3.6	5.8	25
Vestibular kineties, number	3	5	3.8	4.0	0.8	20.8	25
Postoral kineties, number	14	17	15.7	16.0	0.9	5.7	25
Dikinetids in left lateral (dorsal) kinety, number	53	70	62.6	63.0	4.4	7.1	25
Left lateral kineties, number	21	28	24.6	24.0	2.1	8.6	25
Left polykinetid, number	32	36	34.2	34.0	1.1	3.3	25
Left polykinetid length, μm	27	38	33.2	32.7	3.0	9.0	25
Left polykinetid width, μm	9	12	10.0	9.9	0.6	6.1	25
Right polykinetid length, μm	50	68	58.0	55.9	5.5	9.4	25

CV, Coefficient of variation in %; Max, maximum; Mean, arithmetic mean; M, median; Min, minimum; n, number of cells measured; N, number; SD, standard deviation.

**Bryometopia Foissner, 1985**

**Bryometopida Foissner, 1985**

**Bryometopidae Jankowski, 1980**

***Bryometopus*** **Kahl, 1932**

**Figure 4:**
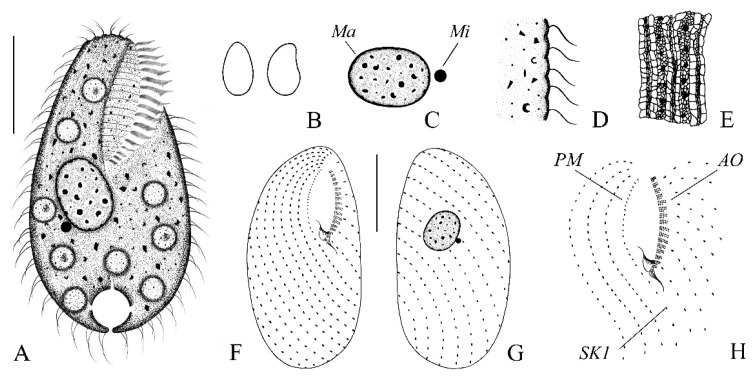
(**A**–**F**) Morphology and ciliary pattern of *Bryometopus shii* n. sp. from life (**A**,**B**,**D**), after protargol staining (**C**,**F**–**H**) and after silver nitrate impregnation (**G**). (**A**) Ventral view of a representative specimen. (**B**) Different body shapes in right lateral view. (**C**) Macronucleus and micronucleus. (**D**) Part of pellicle. (**E**) Silverline system. (**F**,**G**) Ciliature in ventral (**F**) and dorsal (**H**) view of the holotype specimen. (**H**) Ciliature of the oral region. Ma, macronucleus; Mi, micronucleus; PM, paroral membrane; AO, adoral zone of organelles; SK1, somatic kineties 1. Bars, 30 μm (**A**,**F**,**G**).

**Figure 5:**
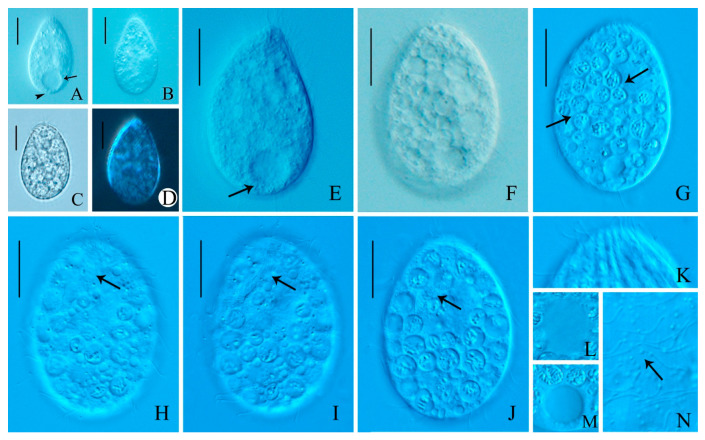
(**A**–**N**) Photomicrographs of *Bryometopus shii* n. sp. from life. (**A**–**J**) General views of different individuals; arrows in (**A**,**E**) show the contractile vacuole; arrows in (**G**) show the food vacuoles; arrows in (**H**–**J**) show the oral apparatus; arrowheads in (**A**) show the excretory pore. (**K**–**N**) Details of the cell (**K**) showing the grooves, serrated pattern in the anterior area, (**L**) the macronucleus, (**M**) the contractile vacuole, (**N**) and the refringent granules (arrows). Bars, 30 μm (**A**–**J**).

**Figure 6:**
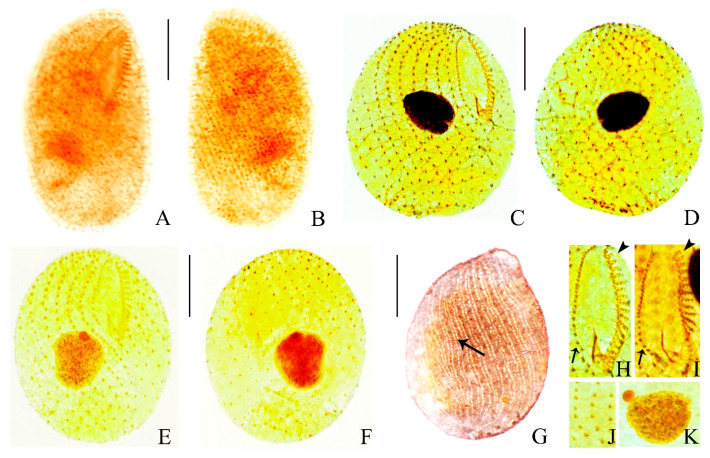
(**A**–**K**) Photomicrographs of *Bryometopus shii* n. sp. after protargol staining (**A**,**B**), silver carbonate-staining and (**C**–**F**,**H**–**K**) dry silver nitrate impregnation (**G**). (**A**,**B**) Ventral and dorsal views of the holotype specimen after protargol staining. (**C**–**F**) Ciliature on ventral and dorsal views of a paratype specimen after silver carbonate-staining. (**G**) Dorsal view of the specimen under dry silver nitrate impregnation; arrow showing the silverline system. (**H**,**I**) Oral apparatus, arrows showing paroral membrane, arrowheads showing adoral zone of organelles. (**J**) Somatic kineties, composed of dikinetids. (**K**) Nuclear apparatus. Bars, 30 μm (**A**–**G**).

***Bryometopus shii*** **n. sp. (Figure 4A–H, Figure 5A–M and Figure 6A–K; Table 2)**

**Diagnosis.** Body 90–105 × 50–70 μm in vivo; droplet-shaped or oval in outline; cytoplasm colorless; single macronucleus; 12–15 somatic kineties; 13–19 adoral zone of organelles; freshwater habitat.

**Type locality.** A puddle near the waste disposal station in Xizha Village, Harbin, Heilongjiang, northeastern China (45°40′27″ N; 126°43′23″ E).

**Figure 7:**
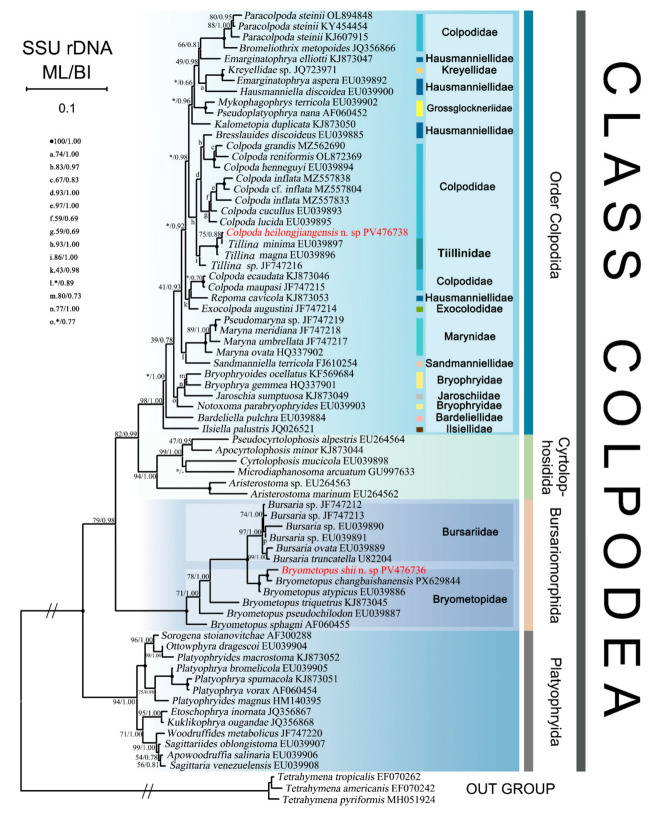
The maximum likelihood (ML) tree based on the SSU-rRNA gene of major members of the class Colpodea, showing the positions of newly sequenced populations of *Colpoda heilongjiangensis* n. sp. and *Bryometopus shii* n. sp. (indicated in red). Node support is shown as ML bootstraps/BI posterior probability. ‘-’ indicates mismatch in topology between Bayesian and ML tree. ‘*’ at nodes indicates support values <30% ML. Fully supported (100%/1.00) branches are marked with solid circles. The long clades have been shortened to approximately 20%, as shown by ‘//’, and the other clades are drawn to scale. Scale bar corresponds to one substitution per 100 nucleotide positions. GenBank accession numbers are listed for all reference sequences.

**Type material.** The protargol slide containing holotype specimen (Figure 5E,F and Figure 7A,B) marked with an ink circle has been deposited in the Laboratory of Protozoology, Ocean University of China (slide number: ZQY–2025072701). A paratype slide (registration number: ZQY–2025072701–02) of protargol-stained specimens is deposited in the Laboratory of Protozoology, Harbin Normal University.

**Etymology.** The species-group name ‘*shii*’ is given in honor of Mr. Shi Xinbai, (Harbin Normal University) in recognition of his great contributions to the protozoology studies.

**Morphology.** Body 90–105 × 50–70 μm in vivo, flexible, ovate or teardrop-shaped in outline and slightly bilaterally flattened (Figure 4A,B and Figure 5A–E). Dorsal side convex, while the ventral side slightly concave, indented along the adoral zone of organelles (Figure 4A and Figure 5A–E). Cytoplasm colorless, laden with many large food vacuoles teeming with bacteria size 7–10 μm (Figure 4A and Figure 5C–G). Contractile vacuole conspicuous, located at the posterior end of the body, ca. 13–15 μm in diameter, pulsating at intervals of 20 s (Figure 4A and Figure 5A,E,M). Cortical granules colorless and transparent, spherical, about 1 μm in diameter, randomly distributed (Figure 5A,B,E,F,N). Rod-shaped excretory pore in center or slightly towards the dorsal side of the posterior pole (Figure 4A and Figure 5A). The cortex features distinct grooves created by somatic kineties, including a serrated pattern in the anterior area (Figure 4A and Figure 5A–K). Macronucleus spherical to oval, located slightly to the left of the cell center, contains many moderately sized, round nucleoli, about 25 × 18 μm (Figure 4A and Figure 5L). Micronucleus located immediately next to macronucleus, about 5 × 4 μm (Figure 4C,G and Figure 6A–F,K). Somatic cilia (approximately 9 μm long) closely arranged around the oral aperture, coursed distinctly spirally posteriorly (Figure 4A and Figure 5A–G). Primarily feeds on bacteria, no green algae observed in vivo (Figure 4A,D and Figure 5A–G,K). Movement moderately fast, lively, usually rotating along the main body axis, and sometimes settling to the bottom and attaching to particles of debris.

In total 12–15 somatic kineties, each composed of paired basal bodies (Figure 4A,F,G, Figure 5E and Figure 6A–F,J). The oral aperture occupied between one-third to one-half of the anterior part of the cell, oriented obliquely to the longitudinal body axis. The shallow, droplet-shaped vestibulum leads to the pharynx. Single lip, located between the adoral zone of organelles and the paroral membrane, borders the right side of the vestibular area. (Figure 4A,F,H and Figure 6A,C,H,I). Paroral membrane aligned alongside somatic kinety 1, exhibited a slight curvature and made up of distinctly angled dikinetids, with ciliation present only on the anterior basal bodies of these structures (Figure 4F,G,H and Figure 6A,C,H,I). Dikinetid in paroral membrane extends into the vestibular trough (Figure 4A,F,G and Figure 6A–F). The oral zone of organelles exhibits a slight curvature, consisting of an average of 13–19 organelles. Each organelle composed of 2 equally long kineties with 4–7 and 1 short kinety with 2–3 basal bodies; short kinety located distally, basal bodies arranged in a zigzag pattern (Figure 4A,F,G,H, Figure 5H–J and Figure 6A,C,H,I). Silverline system composed of both the kreyellid and platyophryid types, tightly and irregularly reticulate (kreyellid) within the ciliary rows and loosely and regularly meshed (platyophryid) between the kineties (Figure 4E and Figure 6G).

**Table 2:** Morphometric characterization of *Bryometopus shii* n. sp. based on protargol-stained specimens.

Character	Min	Max	Mean	M	SD	CV	n
Body length, μm	92	105	96.0	95.0	3.7	3.9	25
Body width, μm	50	65	58.4	59.0	4.7	8.0	25
Macronucleus, number	1	1	1.0	1.0	0.0	0.0	25
Macronucleus length, μm	22	29	25.2	24.9	1.8	7.3	25
Macronucleus width, μm	16	23	18.8	18.3	2.1	11.7	25
Micronucleus, number	1	1	1.0	1.0	0.0	0.0	25
Micronucleus length, μm	4	6	4.9	5.0	0.5	9.7	25
Micronucleus width, μm	3	4	3.3	3.2	0.5	15.7	25
Somatic kineties, number	12	15	13.6	14.0	1.0	7.2	25
Dikinetids in left lateral (dorsal) kinety, number	8	11	9.0	9.0	1.0	11.3	25
Adoral organelles, number	13	19	16.1	16.0	1.9	12.0	25
Adoral dikinetids, number	17	24	20.4	21.0	2.1	10.2	25

CV, Coefficient of variation in %; Max, maximum; Mean, arithmetic mean; M, median; Min, minimum; n, number of cells measured; N, number; SD, standard deviation.

### 3.1. SSU-rRNA Gene Data

The SSU-rRNA gene sequences of *Colpoda heilongjiangensis* n. sp. and *Bryometopus shii* n. sp. have been deposited in the GenBank database with the accession numbers PV476738, PV476736. The length and GC contents are as follows: *Colpoda heilongjiangensisi* (1717 bp, 44.44%) and *Bryometopus shii* (1694 bp, 43.15%).

### 3.2. Phylogenetic Analyses Based on SSU-rRNA Gene Sequences Data

The maximum likelihood (ML) and Bayesian inference (BI) tree had almost identical topologies; therefore, only the ML trees with support values from both methods are presented here (Figure 7). Phylogenetic trees based on SSU-rRNA gene sequences demonstrated the monophyly of all four orders within the class Colpodea (Figure 7). The orders Colpodida Puytorac et al., 1974 and Cyrtolophosidida Foissner, 1978 formed a clade, while Bursariomorphida Fernández-Galiano, 1978 grouped outside this clade. Platyophryida Puytorac et al., 1979 occupied the basal phylogenetic position within Colpodea. The newly sequenced *Colpoda heilongjiangensis* n. sp. formed a sister clade with *C. minima* (EU039897) with moderate support (75% ML/0.88 BI), which subsequently grouped with *C. magna* (EU039896) and *Colpoda* sp. (JF747215).

The clade comprising *Bryometopus shii*, *B. changbaishanensis* and *B. atypicus* clusters with the clade formed by five *Bursaria* species. This combined clade subsequently groups successively with *B. triquetrus*, *B. pseudochilodon*, and *B. sphagni* with moderate support. These results demonstrate the paraphyly of the genus *Bryometopus*.

## 4. Discussion

**Family Colpodidae Bory de St. Vincent, 1826**

**Improved diagnosis.**

Vestibular opening located in the anterior half of the body. Oral cavity ranges from small to large. Right and left oral polykinetids approximately equal in length. Division within reproductive cysts.

**Remarks.**

The family Colpodidea is hereby expanded to incorporate the diagnostic characteristics of former family Tillinidae Foissner, 1985, based on comprehensive morphological analyses. This taxonomic revision unites two groups sharing fundamental ciliary patterns while recognizing distinct morphological specializations.

**Genus** ***Colpoda*** **Müller, 1773**

**Improved diagnosis.**

Larger specimens possess a distinct postoral groove. Vestibulum small to moderately large, funnel-shaped. Left wall of vestibulum overhangs right. Right oral polykinetid composed of few to many short, more or less disordered kineties.

**Remarks.**

Genus *Tillina* Gruber, 1879 (Colpodina group) is formally incorporated into the genus *Colpoda*, with its distinctive *T. magna* morphotype retained as a characteristic subgroup within the genus. The two taxa share homologous LKm fiber systems, with molecular phylogenetic analyses demonstrating the embedded position of the former *Tillina* clade within the *Colpoda* lineage. Further verification through comparative genomics is required to substantiate these findings.

**Comparison of** ***Colpoda heilongjiangensis***  **n. sp. with congeners**

Species of the genus *Colpoda* are mainly characterized by small to large body size, microphagous (mainly bacteria) Colpodidae. Vestibulum small to moderately large, funnel-shaped. Left wall of vestibulum overhangs right. Right oral polykinetid composed of few to many short, more or less disordered kineties. *Colpoda heilongjiangensis* n. sp. is assigned to *Colpoda* based on its large size, single postoral sack, contractile vacuole slightly ahead of posterior end without canals, vestibulum funnel-shaped, left side of vestibulum overhangs right side, left polykinetic row moon-shaped, right oral polykinetid composed of disordered kineties. The following species share a comparable body shape with *Colpoda heilongjiangensis* n. sp. and deserve comparison with this newly described form: *C. minima* Foissner 1993; *C. magna* Lynn 1976; *C. ovinucleata* Foissner 1980; *C. orientalis* Foissner 1993; *C. tripartita* Blatterer & Foissner 1988; *C. lucida* Foissner 1993; *C. cavicola* Foissner 1993; *C. variabilis* Hofmann–Münz et al. 1990 (Table 3).

Three key diagnostic features distinguish *Colpoda heilongjiangensis* n. sp. from *C. minima* and *C. magna*: the lack of a contractile vacuole, a distinctly lower number of somatic kineties, and the presence of a left polykinetid. Specifically, *C. heilongjiangensis* n. sp. differs from *C. minima* in possessing fewer left polykinetids (32–36 vs. 45–65), reduced somatic kineties (56–67 vs. 69–110), and the absence of a contractile vacuole with canals [16,42,43,44,45,46], and it can be further differentiated from *C. magna* by its fewer somatic kineties (56–67 vs. 117–156), fewer left polykinetids (32–36 vs. 75–89), and the complete absence of a canal-bearing contractile vacuole [19,47,48,49,50,51,52].

*Colpoda heilongjiangensis* n. sp. differs from *C. ovinucleata* in having a longer body in vivo (200–260 μm vs. 87–133 μm) and fewer left polykinetids (32–36 vs. 40–70) [53].

*Colpoda heilongjiangensis* n. sp. differs from *C. orientalis* in its larger in vivo body size (200–260 μm vs. 97–112 μm), the absence of a contractile vacuole with collecting canals, and the presence of vestibular kineties, whereas these traits are opposite in *C. orientalis* [16].

*Colpoda heilongjiangensis* n. sp. is morphologically distinct from *C. tripartita* in having a longer body in vivo (200–260 μm vs. 69–100 μm), a higher number of left polykinetids (32–36 vs. 29–32), and a lack of pearl-like cortical granules (vs. present) [54,55].

*Colpoda heilongjiangensis* n. sp. possesses a longer body in vivo (200–260 μm vs. 75–105 μm), more somatic kineties (56–67 vs. 41–55), and an absence of contractile vacuole (vs. present) in comparison with *C. lucida* [16,56,57].

*Colpoda heilongjiangensis* n. sp. is ecologically and morphologically distinct from *C. cavicola* in having a larger body size (200–260 μm vs. 62–170 μm), more somatic kineties (56–67 vs. 38–47), a lack of contractile vacuole with canals (vs. present) and a freshwater habitat (vs. tree-hole dwelling) [16,58,59].

*Colpoda heilongjiangensis* n. sp. is characterized by having a longer body in vivo (200–260 μm vs. 70–156 μm) and missing contractile vacuole canals compared with *C. variabilis* (vs. present) [60,61,62].

**Table 3:** Morphological comparison of *Colpoda heilongjiangensis* n. sp. and their most similar congeners.

Species	Body Length In Vivo, μm	Somatic Kineties, Number	Left Polykinetid, Number	Contractile Vacuole with Canals	Habitat	Reference
***C. heilongjiangensis***	**200–260 × 120–180**	**56–67**	**32–36**	**Absent**	**Freshwater**	**Present work**
*C. minima*	103–315 × 68–207	69–110	45–65	Present	Freshwater/soil	Foissner (1993) [16]
*C. magna*	148–251 × 84–165	117–156	75–89	Present	Freshwater/soil	Lynn (1976) [19]
*C. ovinucleata*	87–133 × 44–70	60–85	40–70	Absent	Pond	Foissner (1980) [53]
*C. orientalis*	97–112 × 55–82	65–76	28–40	Present	Soil	Foissner (1993) [16]
*C. tripartita*	69–100 × 51–83	45–70	29–32	Absent	Moss	Blatterer & Foissner (1988) [52]
*C. lucida*	75–105 × 60–87	41–55	28–37	Present	Soil	Foissner (1993) [16]
*C. cavicola*	62–170 × 55–130	38–47	30–39	Present	Tree-hole	Foissner (1993) [16]
*C. variabilis*	70–156 × 55–113	44–63	37–41	Present	Puddle	Hofmann-Münz et al. 1990 [62]

**Comparison of** ***Bryometopus shii*** **n. sp. with congeners**

Species of the genus *Bryometopus* Kahl, 1932 are mainly characterized as small to large, with shallow vestibulum parallel or oblique to longitudinal axis of cell, paroral membrane extends along right margin of elliptical vestibular trough [16]. *Bryometopus shii* n. sp. is assigned to *Bryometopus* based on vestibulum inclined to the cell’s longitudinal axis, paroral membrane extends into the vestibular trough, adoral zone of organelles slight curvature, ciliary pattern and silverline system. The following six species of Genus *Bryometopus*, which possess shallow vestibulum parallel or oblique to the longitudinal axis of the cell, should be compared in detail with the new species: *B. changbaishanensis* Wang et al., 2016; *B. aypicus* Foissner, 1980; *B. sphagni* Foissner, 1987; *B. pseudochilodon* Foissner, 1993; *B. triquetrus* Foissner, 1993; *B. balantidioides* Foissner, 1993; *B. hawaiien* Foissner, 1994 (Table 4).

*Bryometopus shii* n. sp. can be differentiated from *B. changbaishanensis* by its larger in vivo body size (90–105 μm vs. 40–48 μm), distinct body shape (teardrop-shaped vs. elongate-oval), and divergent habitat preference (freshwater vs. soil) [63].

*Bryometopus shii* n. sp. is readily distinguished from *B. atypicus* by its larger in vivo body size (90–105 μm vs. 45–68 μm), fewer somatic kineties (12–15 vs. 17–23), variable number of kineties per structure (4–7 vs. 5–7), and absence of in vivo green algal endosymbionts (vs. present) [53,64,65].

*Bryometopus shii* n. sp. differs from *B. sphagni* in having fewer somatic kineties (12–15 vs. 43–60) and fewer adoral organelles (13–19 vs. 40–54) [66,67,68].

*Bryometopus shii* n. sp. is morphologically distinct from *B. pseudochilodon* by having a longer body in vivo (90–105 μm vs. 38–55 μm), a lower number of somatic kineties (12–15 vs. 34–42), and fewer adoral organelles (13–19 vs. 22–30) [16,66,69].

*Bryometopus shii* n. sp. is distinguished from *B. triquetrus* by its longer body length in vivo (90–105 μm vs. 43–55 μm), reduced counts of somatic kineties (12–15 vs. 16–20), adoral organelles (13–19 vs. 35–47), and adoral dikinetids (17–24 vs. 35–47) [16,25].

*Bryometopus shii* n. sp. differs from *B. balantidioides* in having a longer body in vivo (90–105 μm vs. 44–65 μm), fewer somatic kineties (12–15 vs. 25–26), adoral organelles (13–19 vs. 25–26), and adoral dikinetids (17–24 vs. 26–30) [16].

*Bryometopus shii* n. sp. can be separated from *B. hawaiiensis* by having a longer body in vivo (90–105 μm vs. 45–63 μm), fewer somatic kineties (12–15 vs. 25–30) and adoral organelles (13–19 vs. 31–42) [70].

**Table 4:** Morphological comparison of *Bryometopus shii* n. sp. and its most similar congeners.

Species	Body Length In Vivo, μm	Somatic Kineties, Number	Adoral Organelles, Number	Adoral Dikinetids, Number	Habitat	Reference
***B. shii***	**90–105 × 50–70**	**12–15**	**13–19**	**17–24**	**Freshwater**	**Present work**
*B. changbaishanensis*	40–48 × 27–34	11–14	11–15	16–26	soil	Wang et al. (2016) [63]
*B. aypicus*	45–68 × 27–34	17–23	17–23	23–30	Soil/Freshwater	Foissner (1980) [60]
*B. sphagni*	77–130 × 52–80	43–60	40–54	Absent	soil	Foissner (1987) [66]
*B. pseudochilodon*	38–55 × 23–31	34–42	22–30	Absent	soil	Foissner (1993) [16]
*B. triquetrus*	43–55 × 24–32	16–20	16–27	35–47	soil	Foissner (1993) [16]
*B. balantidioides*	44–65 × 21–32	25–26	25–26	26–30	soil	Foissner (1993) [16]
*B. hawaiien*	45–63 × 27–37	25–30	31–42	24–36	soil	Foissner (1994) [70]

**Phylogenetic analyses**

Since the 20th century, the taxonomic status of *Tillina* has undergone multiple revisions, reflecting the complex interplay between morphological and molecular evidence. Foissner, through systematic analyses using silver impregnation and scanning electron microscopy [16], demonstrated that taxa previously identified as *Tillina* in earlier studies (e.g., *T. minima* Alekperov 1985 [42]) actually belong to *Colpoda*, thereby relegating *Tillina* to a junior synonym of *Colpoda* [16]. However, Foissner, based on SSU-rRNA gene molecular phylogeny (91% ML/1.00 BI/1.00 PP) and distinctive morphological features (e.g., contractile vacuole with collecting canals; a distinct postoral groove [22]; several to many roof kineties), reclassified *Colpoda magna* and its closely related species into the new family Tillinidae. This taxonomic arrangement attempted to reconcile the contradiction between the high statistical support for molecular clades and the observed morphological divergence.

The new species *Colpoda heilongjiangensis* n. sp. lacks the diagnostic characters of the family Tillinidae (e.g., contractile vacuole with collecting canals; several to many roof kineties) and aligns with the defining features of Colpodidae (e.g., body shape ranges from broad reniform to slender reniform; rod-shaped extrusomes; moon-shaped left polykinetid; right polykinetid composed of disordered kineties). Therefore, it is morphologically classified within the family Colpodidae. In the SSU-rRNA tree, *Colpoda heilongjiangensis* n. sp. clusters with *T. minima* (EU039897) with moderate statistical support (74% ML/0.89 BI). This close relationship is further corroborated by morphological evidence (e.g., single postoral sack; an elliptical macronucleus and a micronucleus; with vestibular kineties; moon-shaped left polykinetid). Although molecular clustering suggests phylogenetic affinity with *Tillina* species, the branch support values fall below the thresholds for establishing independent taxonomic units (ML ≥ 90%/Bayesian posterior probability ≥ 0.95), and significant morphological–molecular conflicts persist. Foissner emphasized that when molecular topological support is insufficient to justify independent taxonomic status [22], while key morphological traits demonstrate unambiguous homology, taxonomic decisions should prioritize maintaining the stability of morphological frameworks [42,45].

Therefore, we propose: (1) reinstating Tillina as a junior synonym of Colpoda, (2) abolishing the family Tillinidae, (3) renaming former Tillinidae species (e.g., *T. minima*, *T. magna*) as *C. minima* and *C. magna*, respectively, and reclassifying them into Colpodidae, and (4) suggesting new family-level diagnostic characteristics, as the morphological criteria for the family Colpodidae have been redefined. The above is merely a preliminary suggestion. However, the present study is only based on the SSU rRNA gene. Definitive conclusions and more comprehensive evidence therefore require further investigations of multi-gene phylogeny.

In the SSU-rRNA gene tree, *Bryometopus shii* n. sp., *B. changbaishanensis* PX629844 and *B. atypicus* (EU039886) group together with full support (100% ML/1.00 BI). This close relationship was also corroborated by morphological similarities (e.g., single macronucleus and micronucleus; rod-shaped excretory pore; cortex features distinct grooves; contractile vacuole located at the posterior end of the body). Although *Bryometopus shii* n. sp. clusters with members of the genus Bursaria with full support (100% ML/1.00 BI), its morphology (e.g., droplet-shaped oral aperture; shallow vestibulum oblique to longitudinal axis of cell; paroral membrane extends to vestibular trough) differs markedly from Bursaria but aligns with the diagnostic features of Bryometopus [16]. Therefore, synthesizing morphological evidence, *Bryometopus shii* n. sp. is retained within genus *Bryometopus*.

The inclusion of the SSU-rRNA gene sequence from *Bryometopus shii* n. sp. failed to resolve the non-monophyly of the genus, which aligns with previous studies [20,23]. This incongruence between molecular and morphological data may be attributed to the rapid evolution of the SSU-rRNA gene and incomplete lineage sorting in ciliates [71,72]. Such conflicts are often reconciled through multi-gene concatenated analyses [73]. To resolve the current taxonomic uncertainty, we recommend that future research re-evaluate the systematic placement of the order Bursariomorphida through expanded taxon sampling and a combined analysis of morphological traits and multi-gene datasets.

To place the new species in a broader framework, members of Colpoda are generally considered cosmopolitan and are often recorded from ephemeral, nutrient-rich microhabitats such as temporary puddles, soil-water interfaces, and organically polluted waters, whereas Bryometopus species are less frequently reported but also tend to occupy similar eutrophic environments [71,72,73]. The new species described herein conform to these generic habitat preferences, as both were isolated from small, organic-rich water bodies with high levels of nutrients and microbial activity, supporting the notion that these genera are ecological generalists within transient aquatic systems. Regarding global distribution patterns, most Colpoda and Bryometopus species have been described from Europe, the Americas, and Asia, but many are known from only single or few localities, reflecting potential undersampling rather than true endemism [71,72,73]. Together, these observations reinforce the ecological distinctiveness of the new species and highlight the importance of continued surveying in under-explored Asian freshwater and soil habitats [64,65,66,67,68,69].

## Data Availability

The information of the two new species in this study has been uploaded to ZooBank. Present work: urn:lsid:zoobank.org:pub: urn:lsid:zoobank. org:pub:75A9A140-104C-41DD-B26D-1F685F1F3DE5. *Colpoda heilongjiangensis* n. sp.: urn:lsid:zoobank.org:act: 20D59124-6611-422A-9BE0-A2E9117204DF. *Bryometopus shii* n. sp.: urn:lsid:zoobank.org:act:151B1807-3C6B-4331-91C0-C215660C1324. The SSU-rRNA gene sequences of the two newly discovered species have been published in GenBank with accession numbers PV476738 and PV476736.

## Abbreviations

Ma	Macronucleus
Mi	Micronucleus
VK	Vestibular kineties
PK	Postoral kineties
rp	Right polykinetid
lp	Left polykinetid
PM	Paroral membrane
AO	Adoral organelles
SK1	Somatic kineties 1
ML	Maximum likelihood
BI	Bayesian inference

## References

[1] Chi Y., Wei F., Tang D., Mu C., Ma H., Wang Z., Al-Rasheid K.A., Hines H.N., Chen X. (2024). Exploring the biogeography, morphology, and phylogeny of the condylostomatid ciliates (Alveolata, Ciliophora, Heterotrichea), with establishment of four new *Condylostoma* species and a revision including redescriptions of five species found in China. Mar. Life Sci. Technol..

[2] Foissner W., Chao A., Katz L.A. (2007). Diversity and geographic distribution of ciliates (Protista: Ciliophora). Biodivers. Conserv..

[3] Jiang L., Wang C., Al-Farraj S.A., Hines H.N., Hu X. (2023). Morphological and molecular examination of the ciliate family Lagynusidae (Protista, Ciliophora, Prostomatea) with descriptions of two new genera and two new species from China. Mar. Life Sci. Technol..

[4] Küppers G.C., da Silva Paiva T., do Nascimento Borges B., Alfaro E.R., Claps M.C. (2019). A new oligotrich (Ciliophora, Oligotrichia) from Argentina, with redefinition of *Novistrombidium* Song and Bradbury. Eur. J. Protistol..

[5] Kabir A.S., Bharti D., Kumar S., Shazib S.U.A., Shin M.K. (2020). Redescription of *Rigidohymena inquieta* (Stokes, 1887) Berger, 2011 as *Metahymena inquieta* gen. nov., comb. nov. (Ciliophora, Hypotricha) based on morphology, morphogenesis, and molecular phylogeny. J. Eukaryot. Microbiol..

[6] Li T., Zhang T., Liu M., Zhang Z., Zhang J., Niu J., Chen X., Al-Farraj S.A., Song W. (2024). Findings on three endocommensal scuticociliates (Protista, Ciliophora) from freshwater mollusks, including their morphology and molecular phylogeny with descriptions of two new species. Mar. Life Sci. Technol..

[7] Liu M., Jiang L., Zhang Z., Wei F., Ma H., Chen Z., Al-Rasheid K.A., Hines H.N., Wang C. (2025). Linking multi-gene and morphological data in the subclass Scuticociliatia (Protista, Ciliophora) with establishment of the new family Homalogastridae fam. nov. Mar. Life Sci. Technol..

[8] Song W., Zhang S., Li Y., Ma H., Li Q., Luo X., Al-Rasheid K.A., Hines H.N., Lu X. (2024). Multi-gene-based investigation on the molecular phylogeny of the hypotrichous family Strongylidiidae (Protista, Ciliophora), with notes on the ontogeny of a new genus and new species. Mar. Life Sci. Technol..

[9] Tang Y., Zheng X., Lu C. (2024). Taxonomy and systematic positions of three new *Callistoctopus* species (Octopoda, Octopodidae) discovered in coastal waters of China. Mar. Life Sci. Technol..

[10] Wu T., Cheng T., Cao X., Jiang Y., Al-Rasheid K.A., Warren A., Wang Z., Lu B. (2023). On four epibiotic peritrichous ciliates (Protozoa, Ciliophora) found in Lake Weishan Wetland: Morphological and molecular data support the establishment of a new genus, *Parapiosoma* gen. nov., and two new species. Mar. Life Sci. Technol..

[11] Small E.B., Lynn D.H. (1981). A new macrosystem for the phylum Ciliophora Doflein, 1901. Biosystems.

[12] Foissner W. (2003). *Pseudomaryna australiensis* nov. gen., nov. spec. and *Colpoda brasiliensis* nov. spec., two new colpodids (Ciliophora, Colpodea) with a mineral envelope. Eur. J. Protistol..

[13] Foissner W. (2010). Life cycle, morphology, ontogenesis, and phylogeny of *Bromeliothrix metopoides* nov. gen., nov. spec., a peculiar ciliate (Protista, Colpodea) from tank bromeliads (Bromeliaceae). Acta Protozool..

[14] Dunthorn M., Eppinger M., Schwarz M.J., Schweikert M., Boenigk J., Katz L.A., Stoeck T. (2009). Phylogenetic placement of the Cyrtolophosididae Stokes, 1888 (Ciliophora; Colpodea) and neotypification of *Aristerostoma marinum* Kahl, 1931. Int. J. Syst. Evol. Microbiol..

[15] Bourland W.A., Vďačný P., Davis M.C., Hampikian G. (2011). Morphology, morphometrics, and molecular characterization of *Bryophrya gemmea* n. sp. (Ciliophora, Colpodea): Implications for the phylogeny and evolutionary scenario for the formation of oral ciliature in the order Colpodida. J. Eukaryot. Microbiol..

[16] Foissner W. (1993). Colpodea (Ciliophora). Protozoenfauna.

[17] Foissner W., de Puytorac P. (1994). Classe des Colpodea Small and Lynn, 1981. Traité de Zoologie, Infusoires Ciliés.

[18] Müller O.F., von Haller A., von Linné C., Bonnet C. (1774). Vermium Terrestrium Et Fluviatilium, Seu Animalium Infusoriorum, Helminthicorum Et Testaceorum, Non Marinorum, Succincta Historia. Vermivm Terrestrium et Fluviatilium, Seu Animalium Infusoriorum, Helminthicorum et Testaceorum, Non Marinorum, Succincta Historia. 1, 2.

[19] Lynn D.H. (1976). Comparative ultrastructure and systematics of the Colpodida. An ultrastructural description of *Colpoda maupasi* Enriquez, 1908. Can. J. Zool..

[20] Li B., Song Y., Hao T., Wang L., Zheng W., Lyu Z., Chen Y., Pan X. (2022). Insights into the phylogeny of the ciliate of class Colpodea based on multigene data. Ecol. Evol..

[21] Song Y., Hao T., Li B., Zheng W., Liu L., Wang L., Chen Y., Pan X. (2022). Study on analysis of several molecular identification methods for ciliates of Colpodea (Protista, Ciliophora). Cell. Microbiol..

[22] Foissner W., Stoeck T., Agatha S., Dunthorn M. (2011). Intraclass evolution and classification of the Colpodea (Ciliophora). J. Eukaryot. Microbiol..

[23] Foissner W., Bourland W.A., Wolf K.W., Stoeck T., Dunthorn M. (2014). New SSU-rDNA sequences for eleven colpodeans (Ciliophora, Colpodea) and description of Apocyrtolophosis nov. gen. Eur. J. Protistol..

[24] Jankowski A. (1980). Conspectus of a new system of the phylum Ciliophora. Tr. Zool. Inst..

[25] Kahl A., Friedrich D. (1932). Urtiere Oder Protozoa I: Wimpertiere Oder Ciliata (Infusoria) 3. Spirotricha.

[26] Jin D., Li C., Chen X., Byerly A., Stover N.A., Zhang T., Shao C., Wang Y. (2023). Comparative genome analysis of three euplotid protists provides insights into the evolution of nanochromosomes in unicellular eukaryotic organisms. Mar. Life Sci. Technol..

[27] Long H., Dong B. (2023). Special topic on EvoDevo: Emerging models and perspectives. Mar. Life Sci. Technol..

[28] Lyu L., Zhang X., Gao Y., Zhang T., Fu J., Stover N.A., Gao F. (2024). From germline genome to highly fragmented somatic genome: Genome-wide DNA rearrangement during the sexual process in ciliated protists. Mar. Life Sci. Technol..

[29] Zhang X., Han W., Fan X., Wang Y., Xu D., Sun K., Wang W., Zhang Y., Ma J., Ye N. (2023). Gene duplication and functional divergence of new genes contributed to the polar acclimation of Antarctic green algae. Mar. Life Sci. Technol..

[30] Ma H., Choi J.K., Song W. (2003). An improved silvercarbonate impregnation for marine ciliated protozoa. Acta Protozool..

[31] Foissner W. (1991). Basic light and scanning electron microscopic methods for taxonomic studies of ciliated protozoa. Eur. J. Protistol..

[32] Wilbert N. (1975). Eine verbesserte technik der protargolimpra gnation fur ciliaten. Mikrokosmos.

[33] Lynn D.H. (2008). The Ciliated protozoa: Characterization, Classification, and Guide to the Literature.

[34] Medlin L., Elwood H.J., Stickel S., Sogin M.L. (1988). The characterization of enzymatically amplified eukaryotic 16S-like rRNA-coding regions. Gene.

[35] Zhang T., Fan X., Gao F., Al-Farraj S.A., El-Serehy H.A., Song W. (2019). Further analyses on the phylogeny of the subclass Scuticociliatia (Protozoa, Ciliophora) based on both nuclear and mitochondrial data. Mol. Phylogenetics Evol..

[36] Hall T.A. (1999). BioEdit: A user-friendly biological sequence alignment editor and analysis program for Windows 95/98/NT. Nucleic Acids Symp. Ser..

[37] Stamatakis A. (2014). RAxML version 8: A tool for phylogenetic analysis and post-analysis of large phylogenies. Bioinformatics.

[38] Ronquist F., Teslenko M., Van Der Mark P., Ayres D.L., Darling A., Höhna S., Larget B., Liu L., Suchard M.A., Huelsenbeck J.P. (2012). MrBayes 3.2: Efficient Bayesian phylogenetic inference and model choice across a large model space. Syst. Biol..

[39] Miller M.A., Pfeiffer W., Schwartz T. (2010). Creating the CIPRES Science Gateway for inference of large phylogenetic trees. Proceedings of the 2010 Gateway Computing Environments Workshop (GCE).

[40] Posada D., Crandall K.A. (1998). MODELTEST: Testing the model of DNA substitution. Bioinformatics.

[41] Kumar S., Stecher G., Tamura K. (2016). MEGA7: Molecular evolutionary genetics analysis version 7.0 for bigger datasets. Mol. Biol. Evol..

[42] Alekperov I.K. (1985). New free-living ciliates from fresh waters of Azerbaijan. Zool. Zh..

[43] Burt R., Kidder G., Claff C. (1941). Nuclear reorganization in the family Colpodidae. J. Morphol..

[44] Dragesco J., Dragesco-Kernéis A. (1986). Ciliés Libres de L’afrique Intertropicale. Introduction à la Connaissance et à L’étude des Ciliés.

[45] Frenkel M. (1982). *Tillina maxgna* (Ciliophora, Colpodidae): Structural changes of the macronucleus correlated with its division. Protistologica.

[46] Díaz S., Martín-González A., Rico D., Gutiérrez J.C. (2003). Morphogenesis of the division and encystment process of the ciliated protozoan *Colpoda minima*. J. Nat. Hist..

[47] Beers C. (1946). *Tillina magna*: Micronuclear number, encystment and vitality in diverse clones; capabilities of amicronucleate races. Biol. Bull..

[48] Gerassimova Z. (1976). The ultrastructure of cortical fibrillar systems in the ciliate *Colpoda steini* and *Tillina magna*. Citologiya.

[49] Gregory L.H. (1909). Observations on the life history of *Tillina magna*. J. Exp. Zool..

[50] Lynn D. (1976). Comparative ultrastructure and systematics of the Colpoda. Fine structural specializations associated with large body size in *Tillina magna* gruber, 1880. Protoistologica.

[51] Foissner V.W. (1985). Klassifikation und Phylogenie der Colpodea (Protozoa: Ciliophora). Arch. Protistenkd..

[52] Foissner W. (1988). Taxonomic and nomenclatural revision of Sládeček’s list of ciliates (Protozoa: Ciliophora) as indicators of water quality. Hydrobiologia.

[53] Foissner W. (1980). Taxonomische studien über die ciliaten des grossglocknergebietes (hohe tauern, österreich). IX. ordnungen heterotrichida und hypotrichida. Ber. Nat. Med. Ver. Salzburg.

[54] Blatterer H. (1989). Beitrag zur terricolen Ciliatenfauna (Protozoa: Ciliophora) Australiens. Stapfia.

[55] Kahl A. (1931). Urtiere oder Protozoa. I. Wimpertiere oder Ciliata (Infusoria). 2. Holotricha. Die Tierwelt Angrenzenden Meeresteile.

[56] Dujardin A. (1841). Histoire Naturelle des Zoophytes: Infusoires.

[57] Gesellschaft zur Beförderung der Gesammten Naturwissenschaften zu Marburg (1927). Sitzungsberichte der Gesellschaft zur Beförderung der Gesammten Naturwissenschaften zu Marburg.

[58] Kahl A. (1935). Urtiere Oder Protozoa: Wimpertiere Oder Ciliata (Infusoria).

[59] Novotny R., Lynn D., Evans F. (1977). *Colpoda spiralis* sp. n., a colpodid ciliate found inhabiting treeholes (Colpodida, Ciliophora). J. Protozool..

[60] Foissner W. (1980). Taxonomische studien uber die ciliaten des grossglocknergebietes (Hohe tauern, Osterreich). VI. familien woodruffiidae, Colpodidae in marynidae. Acta Protozool..

[61] Hofmann-Münz A.H. (1991). The oral apparatus of *Colpoda variabilis* (Ciliophora, Colpodidae): II. Ultrastructure of the oral ciliature and its implications on ciliate phylogeny. Eur. J. Protistol..

[62] Hofmann-Münz A.H., Schoppmann H., Bardele C.F. (1990). The oral apparatus of Colpoda variabilis (Ciliophora, Colpodidae): I. 3-D reconstruction by serial semi-thin sections and low temperature scanning electron microscopy. Eur. J. Protistol..

[63] Wang Y., Wang Y., Li H., Li S., Pan X. (2026). Morphology and molecular phylogeny of two soil ciliate species (Protozoa, Ciliophora) from the Changbai mountain region, China, including a new species. Microorganisms.

[64] Foissner W. (1984). Infraciliatur, Silberliniensystem und Biometrie einiger neuer und wenig bekannter terrestrischer, limnischer und mariner Ciliaten. Stapfia.

[65] Wirnsberger E., Foissner W., Adam H. (1985). Morphogenesis, fine structure and phylogenetic relationships of the heterotrich ciliate *Bryometopus atypicus* (Protozoa, Colpodea). Ann. Sci. Naturelles. Zool. Biol. Anim..

[66] Foissner W. (1987). Neue und wenig bekannte hypotriche und colpodide Ciliaten (Protozoa: Ciliophora) aus Böden und Moosen. Zool. Beitr..

[67] Penard E. (1922). Etudes sur les Infusoires D’eau Douc.

[68] Reuter J. (1961). Einige faunistische und ökologische Beobachtungen über Felsentümpel-Ziliaten. Acta Zool. Fenn..

[69] Foissner W., Adam H., Foissner I. (1982). Morphologie und Infrastruktur von *Bryometopus pseudochilodon* KAHL, 1932, *Balantidiodes dragescoi* nov. spec. und *Kabiella marina* nov. spec. und Revision des Genus Balantidioides PENARD, 1930 (Protozoa, Ciliophora). Protistologica.

[70] Foissner W. (1994). *Bryometopus hawaiiensis* sp. n., a new colpodid ciliate from a terrestrial biotope of the Hawaiian archipelago (Protozoa: Ciliophora). Ann. Naturhistorischen Mus. Wien. Ser. B Bot. Zool..

[71] Bernhard D., Stechmann A., Foissner W., Ammermann D., Hehn M., Schlegel M. (2001). Phylogenetic relationships within the class Spirotrichea (Ciliophora) inferred from small subunit rRNA gene sequences. Mol. Phylogenetics Evol..

[72] Gentekaki E., Kolisko M., Boscaro V., Bright K., Dini F., Di Giuseppe G., Gong Y., Miceli C., Modeo L., Molestina R. (2014). Large-scale phylogenomic analysis reveals the phylogenetic position of the problematic taxon Protocruzia and unravels the deep phylogenetic affinities of the ciliate lineages. Mol. Phylogenetics Evol..

[73] Dunthorn M., Otto J., Berger S.A., Stamatakis A., Mahé F., Romac S., de Vargas C., Audic S., Consortium B., Stock A. (2014). Placing environmental next-generation sequencing amplicons from microbial eukaryotes into a phylogenetic context. Mol. Biol. Evol..

